# Analysis of outer membrane vesicle associated proteins isolated from the plant pathogenic bacterium *Xanthomonas campestris *pv. campestris

**DOI:** 10.1186/1471-2180-8-87

**Published:** 2008-06-02

**Authors:** Vishaldeep K Sidhu, Frank-Jörg Vorhölter, Karsten Niehaus, Steven A Watt

**Affiliations:** 1 Dep.7 Proteome and Metabolome Research, Faculty of Biology, Bielefeld University, POB 10 01 31, D-33501 Bielefeld, Germany; 2 Center for Biotechnology, Bielefeld University, Universitaetstr. 25, 33615, Bielefeld, Germany

## Abstract

**Background:**

Outer membrane vesicles (OMVs) are released from the outer membrane of many Gram-negative bacteria. These extracellular compartments are known to transport compounds involved in cell-cell signalling as well as virulence associated proteins, e.g. the cytolysine from enterotoxic *E. coli*.

**Results:**

We have demonstrated that *Xanthomonas campestris *pv. campestris (*Xcc*) releases OMVs into the culture supernatant during growth. A proteome study identified 31 different proteins that associate with the OMV fraction of which half are virulence-associated. A comparison with the most abundant outer membrane (OM) proteins revealed that some proteins are enriched in the OMV fraction. This may be connected to differences in the LPS composition between the OMVs and the OM. Furthermore, a comparison of the OMV proteomes from two different culture media indicated that the culture conditions have an impact on the protein composition. Interestingly, the proteins that are common to both culture conditions are mainly involved in virulence.

**Conclusion:**

Outer membrane vesicles released from the OM of *Xcc *contain membrane- and virulence-associated proteins. Future experiments will prove whether these structures can serve as "vehicles" for the transport of virulence factors into the host membrane.

## Background

The Gram-negative bacterium *Xanthomonas campestris *pv. campestris (*Xcc*) is the causal agent of "black rot" disease in crucifers, which include a number of plants of economical importance. *Xanthomonas campestris *can be subdivided into different pathovars according to their host range [1,2]. Xanthomonads invade compatible host plants via hydathodes, stomata or wound openings. The infection process itself is facilitated by high temperatures and humidity, which are climatic characteristics of a multitude of developing countries in Latin America, Africa and Asia, where *Xanthomonas *epidemics account for substantial economical losses [2]. To gather a deeper understanding of how these bacteria cause disease in different plants, the genome sequences of four different *Xanthomonas *pathovars were established [3-5]. Furthermore, recent sequencing projects have revealed the genomic information of three different *Xanthomonas campestris *pv. campestris strains [3,6,7]. This plethora of genomic information is a useful resource to identify genes involved in host specific pathogenicity and in more general virulence mechanisms. The specific host range determining mechanisms are orchestrated by the fine tuned interaction of pathogen derived effector proteins and structures within the host cell [8,9]. These proteins are commonly directly delivered into the host cell by the type-III secretion system (TTSS), which is highly conserved among Gram-negative pathogens. The components of the TTSS are usually encoded by the so-called *hrp*-operon (hypersensitive response and pathogenicity), which consists of approximately 20 genes, of which nine are highly conserved and therefore termed *hrc *[9]. The transcription of the *hrp*-genes is tightly controlled and only activated in environments that resemble the host [10]. One of the key proteins of the TTSS is the HrpF pore, which is inserted into the host's plasma membrane and enables the pathogen to channel effector proteins into the host cell [11,12]. General virulence mechanisms involve proteins responsible for trace element acquisition (e.g., iron), extracellular proteins with lytic functions as well as protein complexes involved in their secretion [13-15]. These proteins aid the colonization of the host and a saprophytic lifestyle in later stages of disease.

Only recently, the role of outer membrane derived vesicles, so-called outer membrane vesicles (OMVs), have been studied in detail. They have been found to act as vehicles for the transport of virulence associated compounds into other cells [16-18]. OMVs are known to be constantly liberated from the outer membrane of most Gram-negative bacteria [19]. It has been demonstrated that they contain outer membrane and periplasmic proteins and in some cases DNA or cell-cell signalling molecules [20,21]. This makes OMVs an ideal structure to transport hydrophobic compounds like membrane proteins into the host. The best-studied membrane anchored virulence factor is the ClyA protein from enterotoxic bacterium *E. coli*, which forms pores inside the host membrane and thus causes lysis. It has been demonstrated that ClyA is inserted into the outer membrane of the pathogen as inactive monomers, which are then released and embedded in OMVs where they are converted into an active form that assembles into the multimeric membrane pore. Upon fusion of the OMV with the host membrane a functional ClyA pore is inserted [16]. This clearly indicates the potential of OMVs to deliver membrane active virulence factors into the host.

In this study, we present the proteome of OMVs isolated from the culture supernatant of the *Xcc *strain B100. Furthermore, we could demonstrate that the composition of the OMV proteome differs from that of the outer membrane implying some kind of protein sorting mechanism. A considerable amount of proteins identified in the OMV fraction are assigned virulence factors or are connected to effector protein transport indicative of the probable involvement of OMVs as vehicles for these compounds.

## Results

### OMVs are released from the outer membrane of *Xcc *during growth

**Figure 1:**
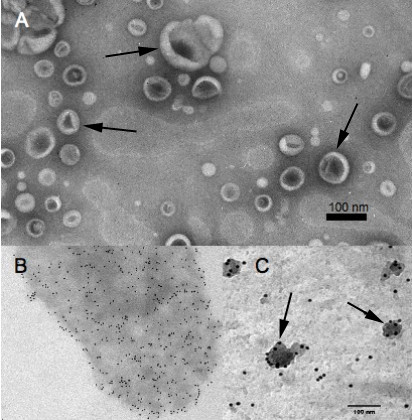
**Electron micrgraphes of *Xcc *cell and OMV preparations**. (A) Electron micrograph of 1% (w/v) uranyl acetate stained outer membrane vesicles extracted from the cell free supernatant of *Xanthomonas campestris *pv campestris strain B100 at a 30 k fold magnification (OMVs highlighted by arrows). (B) Immunogold electron microscopy of 1% (w/v) uranyl acetate stained *Xcc *cell at 14 k fold magnification after incubation with an anti-*Xcc*B100 antibody which was detected by a 10 nm gold particle bound to a goat anti-rabbit antibody (gold-labelled OMV highlighted by arrows). (C) Immunogold electron micrograph of 1% (w/v) uranyl acetate stained OMVs at 27 k fold magnification after incubation with an anti-*Xcc*B100 antibody detected with a 10 nm gold particle bound to an goat anti-rabbit antibody.

**Figure 2:**
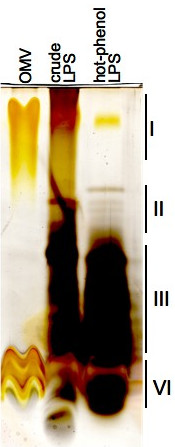
**SDS-PAGE performed with LPS from OMVs and whole cell preparations**. The OMVs as well as a crude and a hot-phenol purified LPS preparation were separated on an SDS-PAGE and stained with an LPS-specific silver stain. The gel displays different bands resembling: (I) mature LPS consisting of the O-antigen, the core region attached to lipid-A; (II) LPS biosynthesis intermediates with incomplete O-antigen; (III) Lipid A attached to the core region; (VI) free lipid-A structure.

Electron microscopy of an OMV fraction isolated from the culture supernatant of M9 minimal medium was performed to determine whether *Xcc *releases OMVs into the medium and to test the applicability of the purification method. The electron micrographs of negatively stained outer membrane vesicles mounted on glow-discharged carbon-coated nickel grids indicate that most vesicles have a spherical shape. A few dented vesicles can also be observed (Fig. 1A). The dents may be a result of dehydration, which occurs during the preparation of the vesicles. The vesicle diameters range from 10 to 100 nm with an average diameter of 45 ± 21 nm (n = 373). Immunogold-labeling of whole cell and vesicle preparations was performed using an anti-*Xcc *antibody [22]. As displayed in Fig. 1B and 1C, the antibody specifically recognized the *Xcc *cells and thus *Xanthomonas *surface-derived compounds in the OMVs. Furthermore, a lipopolysaccharide (LPS)-specific SDS-PAGE was performed to compare the LPS composition of whole cell preparations to that of the OMVs (Fig. 2). The lanes on the gel loaded with the crude LPS preparation and the hot-phenol purified sample displayed bands representing LPS (consisting of O-antigen, the core region and lipid-A), the core attached to lipid-A and free lipid-A. The bands located between the core and LPS represent LPS synthesis intermediates consisting of the core and different amounts of oligo-saccaride subunits. The lane loaded with the OMV sample only displays a band representing the mature LPS structure and free lipid-A. This indicates that the OMVs are derived from the outer membrane and more interestingly only contain mature LPS whilst the whole cell preparations also contain intermediates of the LPS biosynthesis.

### The OMV proteome of *Xcc *contains outer membrane and virulence-associated proteins

**Figure 3:**
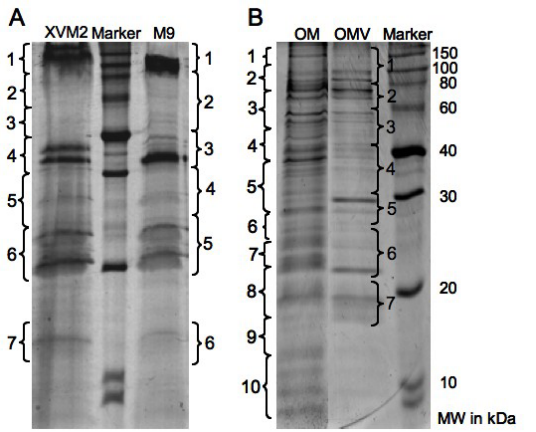
**SDS-PAGE of proteins extracted from OMVs of different culture conditions and the outer membrane**. (A) A 12% acrylamide SDS-PAGE performed with proteins obtained from OMVs of *Xcc *cultures grown in M9 and XVM2 medium. The brackets with numeration indicate the sections taken from the gel for tryptic digestion. (B) Protein profiles of OMVs isolated from cultures grown in M9 medium and the outer membrane (OM) fraction prepared from the same culture.

**Figure 4:**
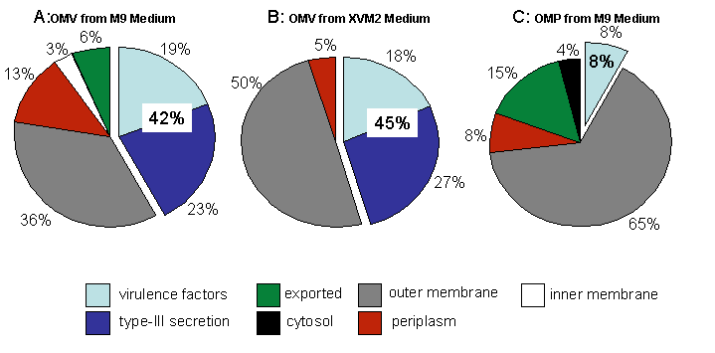
**Pie charts of the protein groups found in the OMVand the OM preparations**. The proteins identified from OMVs and OMPs were sub-divided into seven groups according to their proposed sub-cellular location or function. The pie charts indicate the number of proteins in each group isolated from OMVs collected from M9 medium (A) from XVM2 medium (B) and the outer membrane proteins isolated from cells grown in M9 medium (C). The percentage values within the pie chart give the amount of proteins grouped as virulence-associated, which comprise the groups of virulence factors and type III secretion proteins.

To analyse its protein cargo, the OMV fraction was harvested from the cell-free culture supernatant of M9 cultures and subjected to SDS-PAGE. Previous attempts to resolve the OMV protein fraction using two-dimonesional gel electrophoresis failed due to the high amount of exopolysaccharides that are co-purified with the vesicles. Another problem was the low amount of proteins (30 μg·mL^-1^) that could be purified from o litre of culture supernatant, which prohibited further purification steps. Therefore, a combination of SDS-PAGE and nano-LC-ESI-MS/MS of tryptically-digested gel-sections was employed to identify the proteins that associate with the OMV fraction. This method has been described previously for various membrane proteome studies [42,43]. The resulting Coomassie stained SDS-PAGE revealed approximately 15 protein bands, which were excised in vertical sections comprising 2–6 protein bands per section for subsequent trypsin digestion (Fig. 3A). The trypsin-generated peptide masses, as well as their fragment ions were determined by nano-LC-ESI-MS/MS. This data was the basis for a MASCOT aided database query, which led to the identification of 31 different proteins (Table 1 additional MS/MS data is presented in Table 4 in Additional file 1). A query result was only considered as significant if the overall score was higher than 25, more than two tryptic peptides as well as their fragment ions matched to the protein in question and the calculated molecular weight fitted to the initial gel section. To verify the ESI-MS/MS results, individual protein bands were excised from the SDS-PAGE and treated as already described. The tryptic peptides were analysed using a MALDI-TOF-MS approach and the resulting peptide mass fingerprints were used for a MASCOT aided database query. In this approach, only scores higher that 49 were considered significant. Only seven proteins could not be verified using the MALDI-TOF-MS approach (FadL, Ffh, AvrBs2, XynB, RlpA, RlpB, Lipoprotein precursor). They can also be considered as "insecure" candidates also because their MASCOT-significance scores are close the threshold (Table 1). Three proteins, HrcU, OmpA and OmpW3, could be identified in more than one gel section. Since they were in neighbouring sections, it can be assumed that they originate from protein bands, which were located in the section border. According to the function or cellular localization it was possible to cluster the proteins into 7 groups (Fig. 4). Five of these groups consider the localization of the proteins within the cell and were termed "cytosol", "inner membrane", "periplasm", "outer membrane" and "exported". The remaining two groups harbour proteins involved in virulence, including "virulence factors" and proteins of the "type-III secretion" system (Fig. 4A, B). The two major protein groups in the OMV proteome of M9 cultivated *Xcc *cells associate with the "outer membrane" (36%), its majority being TonB-dependent receptor proteins, and the virulence-associated group (42%), which consists of "virulence factors" and "type-III associated" proteins. The virulence-associated group does not only consist of the membrane attached Hrc, Hrp (23%) and HrpF proteins but also of "virulence factors" (19%) consisting of avirulence proteins and two cell wall degrading enzymes, a cellulase (Egl) and a xylosidase (XynB). Furthermore, the OMVs contain 13% periplasmic proteins, which may be entrapped in the vesicle lumen during their releases from the outer membrane. A software-aided analysis of the C-termini of all identified proteins revealed that 39% have a putative secretion signal, which makes them targets for the general secretion pathway.

**Table 1:** Proteins identified in the OMV fraction of cultures grown in M9 medium.

**Section**	**Protein**	**Acc. No.**	**MW(cal)**	**Size range**	**Coverage (ESI-MS/MS)**	**Score (ESI-MS/MS)**	**Coverage (MALDI)**	**Score (MALDI)**
1	TonB-dependent receptor with signalP	AAM 42139	100858	80–150	10	162	31	134
	Outer membrane protein With signalP	AAM40663	90600		6	113	27	96
	**Oar **putative outer membrane receptor protein, tonB-dependent with signalP	AAM41771	117723		4	91	11	86
	TonB-dependent outer membrane receptor	AAM41663	91909		6	49	9	89
	**FpvA **TonB-dependent outer membrane ferripyoverdine receptor with signalP	AAM42628	81083		6	59	8	60
	**HrcV **(HrcV)	AAM40527	69015		9	32	12	54
	**HrpF **(HrpF protein)	AAM40515	98836		11	30	19	92
	**AcrD **Outer membrane efflux protein	AAM41427	130193		8	27	11	102

2	**FadL **outer membrane fatty acid porin with signalP	AAM39336	47455	60–80	13	235	-	-
	**Egl **exported cellulase with signalP	AAM42791	52209		7	140	18	88
	Avirulence protein with signalP	AAM43445	67281		5	27	9	59
	**HrcN **HrcN protein	AAM40534	47759		5	30	7	52
	**ffh **Signal recognition particle protein	AAM40492	49312		7	27	-	-
	**AvrBs2 **(avirulence protein)	AAM39371	78471		3	27	-	-

3	**OmpA **family outer membrane protein with signalP	AAM40245	39338	40–60	27	405	21	143
	**AvrBs1 **avirulence protein AvrBs1	AAM41388	49789		6	29	8	61
	**XynB **exported xylan 1,4-beta-xylosidase with signalP	AAM43196	58311		3	32	-	-
	**HrcU **HrcU protein	AAM40528	38694		6	27	6	57

4	**OmpA **family outer membrane protein with signalP	AAM40245	39338	30–40	32	402	19	96
	**VirB6 **protein	AAM42571	38386		6	32	5	56
	**Hrc U **HrcU protein	AAM40528	38694		8	30	8	58
	**RlpA **Exported rare lipoprotein A With signalP	AAM42727	46235		6	35	-	-
	HrpW* (HrpW protein)	AAM40517	33372		2	28	11	59

5	Putative exported protein	AAM40085	35080	25–30	8	53	9	68
	**OmpW3 **OmpW family outer membrane protein	AAM42791	23494		7	41	10	63
	*RlpB Rare lipoprotein B with signalP	AAM40532	22959		10	31	-	-
	Lipoprotein precursor with signalP	AAM41856	26209		4	26	-	-

6	**OmpW3 **OmpW family outer membrane protein	AAM42791	23494	20–25	8	58	10	72
	**HrpB4 **HrpB4 protein	AAM40532	22919		4	29	4	52
	**HpaH **Putative transglycosylase HpaH	AAM40539	21109		3	29	6	58

7	Putative exported protein with signalP	AAM40594	10647	15–20	15	171	22	127
	**XpsH **General secretion pathway protein	AAM39979	18208		20	47	17	61
	**UptD **outer membrane protein with signalP	AAM39910	17116		15	39	18	83
	**HrpE **HrpE protein	AAM40519	9749		30	30	32	56

The section indicates the gel-section excised from the corresponding SDS-PAGE. The values indicated under MW(cal) gives the calculated molecular weight of the identified proteins. The size range relates the excised gel-section with regard to the molecular weight standard on the SDS-PAGE. Score (MOWSE-score) and coverage (sequence coverage) relate to the two approaches (ESI-MS/MS or MALDI-TOF-MS) utilized for the identification of the proteins on the SDS-PAGE.

In summary, the proteome of the OMVs mainly consists of proteins associated to the outer membrane and proteins either located in the periplasm or travelling through it.

### OMVs do not contain all high abundant proteins present in the OM proteome

A comparison of the proteins extracted from OMVs and the outer membrane (OM) was conducted to analyse if there is a preference for certain proteins to be located in the OMVs. The outer membrane fraction was also separated by SDS-PAGE and sectioned as already described (Fig. 3B). Each gel section was digested with trypsin and analysed by nano-LC-ESI-MS/MS. The comparison of the SDS-PAGE patterns of the OMV and OM proteome revealed a clear difference between the two proteomes (Fig. 3B). The MASCOT software-aided database query using the ESI-MS/MS data resulted in the significant identification of 26 different proteins (Table 2 additional MS/MS data is presented in Table 5 in Additional file 1). This number clearly does not reflect the exact number of proteins present in the OM fraction but rather stands for the most abundant proteins in each gel section. The OM protein preparation contains 15 different outer membrane proteins, most of them being TonB-dependent receptors (9). Since most of these 15 proteins have probability scores above 200 it can be presumed, that they belong to the group of high abundant outer membrane proteins. Interestingly, only half of these proteins are also present in the OMV protein fraction. There are five further outer membrane proteins with probability scores greater than 140 that can only be identified in the OM proteome. Furthermore, only three of the nine TonB-dependent receptors can be found in both protein preparations. The OM protein fraction revealed 11 non-outer membrane proteins, among those are six secreted, four inner membrane-associated and one cytosolic (TufA) protein. The inner membrane-associated proteins are co-purified with the outer membrane, since the ultrasonification process will also liberate inner membrane fragments. The secreted proteins could be co-purified whilst they are associated with one of the membranes during the secretion process. Among this class of non-outer membrane proteins are three that could be identified in the OM and OMV fraction alike (Egl, Ffh, XpsH). Only two proteins, a cellulase (Egl) and a β-glucosidase (Bgl), could be assigned as virulence factors. Surprisingly, no type-III secretion-associated proteins, which are present in abundance in the OMV proteome, are among the identified in the OM fraction.

**Table 2:** Proteins identified in the outer membrane fraction of cultures grown in M9 medium.

**Section**	**Protein**	**Acc.No.**	**MW(cal)**	**Size range**	**Coverage (ESI-MS/MS)**	**Score (ESI-MS/MS)**	**Coverage (MALDI)**	**Score (MALDI)**
1	TonB-dependent outer membrane receptor with signalP	AAM 42139	100858	100–150	19	664	114	16
	**Oar **putative outer membrane receptor protein, tonB-dependent with signalP	AAM41771	117723		10	295	64	12
	TonB-dependent outer membrane receptor (C-terminal fragment) with signalP	AAM43353	110414		8	271	65	6
	TonB-dependent outer membrane receptor (C-terminal fragment) with signalP	AAM42431	106797		7	141	-	-
	TonB-dependent outer membrane receptor with signalP	AAM42675	103487		3	53	57	5

2	TonB-dependent outer membrane receptor with signalP	AAM42139	100858	80–100	14	630	124	18
	TonB-dependent outer membrane receptor with signalP	AAM42044	83577		21	628	-	-
	**FpvA **TonB-dependent ferripyoverdine receptor precursor with signalP	AAM42628	81083		20	506	116	16
	**PhuR **outer membrane hemin receptor, tonB-dependent with signalP	AAM41930	85127		17	317	92	13
	TonB-dependent outer membrane receptor with signalP	AAM42316	84374		8	218	64	8
	Outer membrane protein with signalP	AAM40663	90600		6	113	72	8
	**Bgl **exported beta-glucosidase with signalP	AAM41065	91830		4	34	-	-

3	**Egl **exported cellulose with signalP	AAM42791	52209	50–80	7	142	68	6
	OmpA family outer membrane protein with signalP	AAM40245	39338		8	98	72	16
	**Ffh **Signal recognition particle protein	AAM40492	49312		6	31	-	-

4	**OmpA **family outer membrane protein with signalP	AAM40245	39338	40–50	34	581	18	93
	**FadL **outer membrane fatty acid porin with signalP	AAM39336	47455		11	229	-	-
	**TufA **(elongation factor Tu)	AAM40191	43181		14	70	88	12

5	**OmpA **family outer membrane protein with signalP	AAM40245	39338	30–40	32	402	73	16
	Putative secreted protein	AAM39862	29865		5	41	63	9
	**Egl **exported cellulose	AAM42791	52209		8	39	55	6
	**XpsE **Type II secretory pathway ATPase	AAM39976	64291		3	29	-	-
	**FliC **Flagellin A (Flagellin core protein)	AAM41230	40380		3	29	64	8

6	**OmpA **family outer membrane protein with signalP	AAM40245	39338	27–30	29	515	92	18
	Putative secreted protein	AAM39862	29865		3	40	56	8
	**FadL **(outer membrane fatty acid porin)	AAM39336	47114		3	26	-	-

7	**OmpA **family outer membrane protein with signalP	AAM40245	39338	24–27	9	88	73	16
	Putative exported protein	AAM40085	35080		8	60	63	12
	**UptE **Outer membrane protein with signalP	AAM39911	31591		26	37	73	32

8	**OmpW3 **ompW family outer membrane protein	AAM39855	23494	20–24	7	55	58	13
	**XpsH **General secretion pathway protein	AAM39979	18208		11	47	63	21
	Putative secreted protein	AAM39862	29865		7	26	74	14

9	**UptD **outer membrane protein with signalP	AAM39910	17116	15–20	13	57	60	14
	**WxcE **exported protein	AAM39921	20490		11	26	54	9

10	Putative exported protein with signalP	AAM40594	10647	10–15	15	117	-	-
	**UptD **outer membrane protein with signalP	AAM39910	17116		6	33	64	16

The section indicates the gel-section excised from the corresponding SDS-PAGE. The values indicated under MW(cal) gives the calculated molecular weight of the identified proteins. The size range relates the excised gel-section with regard to the molecular weight standard on the SDS-PAGE. Score (MOWSE-score) and coverage (sequence coverage) relate to the two approaches (ESI-MS/MS or MALDI-TOF-MS) utilized for the identification of the proteins on the SDS-PAGE.

Although the OMVs originate from the outer membrane only less than half of the most abundant OM proteins could also be identified in the OMV fraction. Noteworthy is that all but one of the virulence associated proteins of the OMVs are among the high abundant proteins of the outer membrane. Therefore, 21 proteins, most of them virulence-associated, seem to be enriched in the OMV fraction.

### The growth medium has an influence on the OMV protein composition

The XVM2 medium, which is known to induce genes coding for the type-III secretion system and virulence factors [27], was used to study the influence of growth conditions on the OMV proteome. The OMV proteins isolated from XVM2 cultures were treated as already described. This procedure led to the identification of 23 different proteins using the LC-ESI-MS/MS approach (Table 3 additional MS/MS data is presented in Table 6 in Additional file 1). The MALDI-TOF-MS approach could verify the identity of all but five proteins (XadA1, AvrBs1, FasD, FadL, XynB). These proteins can be regarded as "insecure" identifications. Nonetheless, 50% of the identified proteins belong to the group "outer membrane". Most (9) of them are also present in the proteomes of the OM and OMVs isolated from M9 cultures. Furthermore, seven proteins could only be identified in the OMV fractions isolated from both culture conditions; these include three type-III secretion-associated proteins (HrpF, HrcU, HrpB4), three virulence factors (AvrBs2, AvrBs1, XynB) and only one additional protein that is not involved in virulence (XpsH). Another 6 proteins are unique to OMVs of the XVM2 culture. One of these proteins is HrpXv, the regulator of the type-III secretion system, which might be induced by the culture condition. How this protein associates with the OMV fraction, however, is unclear. A noteworthy protein, which also belongs to the unique proteins, is the adhesion protein XadA1, which might be co-regulated with the *hrp*-regulon. The OMVs isolated from M9 medium contain 14 unique proteins. Interestingly, 4 of these proteins belong to the type-III secretion system (HrcV, HrcN, HrpW, HrpE), which should not be induced under this culture condition. Also noteworthy are 3 lipoproteins, which are exclusive to OMVs from the M9 culture. This indicates that the growth medium has a clear influence on the protein composition of the OMVs. However, 9 proteins can be regarded as the core-proteome. They reflect the outer membrane origin of the OMVs, since they are also among the most abundant OM proteins. The 7 proteins common to OMVs isolated from both media seem to be characteristic for the OMV proteome and a majority of them are involved in virulence.

**Table 3:** Proteins identified in the OMV fraction of cultures grown in XVM2 medium.

**Section**	**Protein**	**Acc.No.**	**MW(cal)**	**Size range**	**Coverage (ESI-MS/MS)**	**Score (ESI-MS/MS)**	**Coverage (MALDI)**	**Score (MALDI)**
1	TonB-dependent receptor with signalP	AAM 42139	100858	80–150	8	311	10	134
	**XadA1 **Xanthomonas adhesion XadA	AAM39974	220132		7	35	-	-
	**HrpA **ATP-dependent RNA helicase	AAM42217	151602		6	32	8	92
	**RhsD **(RhsD protein) with signalP	AAM39448	164320		5	32	7	66

2	**FpvA **TonB-dependent outer membrane ferripyoverdine receptor with signalP	AAM42628	81083	60–100	9	162	12	118
	TonB-dependent outer membrane receptor	AAM41663	91909		4	61	8	86
	**HrpF **(HrpF protein)	AAM40515	98836		6	35	14	84
	**AvrBs2 **(avirulence protein)	AAM39371	78471		10	33	-	-
	**FasD **outer membrane usher protein	AAM40677	86678		6	30	-	-

3	**OmpA **family outer membrane protein with signalP	AAM40245	39338	40–60	28	505	21	97
	**XynB **exported xylan 1,4-beta-xylosidase with signalP	AAM43196	58311		5	34	-	-
	**UptE **Outer membrane protein with signalP	AAM39911	31591		15	33	19	56
	**AvrBs1 **avirulence protein AvrBs1	AAM41388	49789		8	31	13	68

4	**OmpA **family outer membrane protein with signalP	AAM40245	39338	30–40	18	113	20	102
	**Egl **exported cellulase with signalP	AAM42791	52209		4	39	9	86
	**HrcU **HrcU protein	AAM40528	38694		8	30	12	57
	**HrpX **AraC-type transcriptional regulator HrpX	AAM40465	52250		6	30	18	83
	**HrcN **HrcN protein	AAM40534	47759		12	29	10	58

5	**OmpW3 **OmpW family outer membrane protein	AAM42791	23494	25–30	7	55	7	67
	**HrpB4 **HrpB4 protein	AAM40532	22919		4	36	8	59

6	**OmpW3 **OmpW family outer membrane protein	AAM42791	23494	20–25	7	48	7	71
	**XpsH **General secretion pathway protein	AAM39979	18208		20	47	17	61

7	Putative exported protein with signalP	AAM40594	10647	10–20	15	245	21	117
	**UptD **outer membrane protein with signalP	AAM39910	17116		12	27	17	74

The section indicates the gel-section excised from the corresponding SDS-PAGE. The values indicated under MW(cal) gives the calculated molecular weight of the identified proteins. The size range relates the excised gel-section with regard to the molecular weight standard on the SDS-PAGE. Score (MOWSE-score) and coverage (sequence coverage) relate to the two approaches (ESI-MS/MS or MALDI-TOF-MS) utilized for the identification of the proteins on the SDS-PAGE.

## Discussion

### Outer membrane vesicles are released from the bacterial surface and contribute membrane proteins to the extracellular proteome

Previous work of our group describes a comprehensive study of the extracellular proteome of *Xanthomonas campestris *pv. campestris. There we identified some outer membrane proteins among the most abundant extracellular proteins (OmpA family protein AAM40245; OmpW AAM39855; TonB-dependent receptor AAM42139) [16]. Since outer membrane vesicles (OMVs) are known to be constantly liberated from the outer membrane of Gram-negative bacteria, we suspected that OMVs could be the source of outer membrane proteins in the culture supernatant [23,19]. Thus we first employed electron microscopy to demonstrate that *Xanthomonas campestris *pv. campestris strain B100 releases outer membrane vesicles. The OMV proteome revealed that the outer membrane proteins found in the extracellular proteome are also present here. Furthermore, two other proteins could be identified in both proteomes, the cellulase Egl (AAM42791) and a putative secreted protein (AAM40085). This indicates that OMVs contribute to the extracellular proteome by releasing outer membrane proteins into the environment. Outer membrane vesicles of other Gram-negative bacteria have been described to contain outer membrane proteins, periplasmic proteins, lipopolysaccharides, phospholipids, DNA, toxins and other factors associated with virulence [23,20,21]. Previous studies have demonstrated that bacterial OMVs can interact with membranes of other Gram-negative and Gram-positive bacteria, which can result in lysis of the target organism [25]. Furthermore, Wai and co-workers [16] could demonstrate that OMVs can even deliver toxins into the membrane of a eukaryotic host cell.

Electron micrographs demonstrate that vesicles produced by *Xcc *are spherical with a diameter ranging between 10 and 100 nm with a mean diameter of 45 nm. These OMVs are much smaller than those described for mammalian pathogens, e.g., enterotoxic *E. coli *or *Helicobacter pylori*, which have diameters ranging up to 300 nm [26,19]. The reduced OMV size could be an adaptation towards the plant cell wall, which does not permit the passage of large structures. Immunogold electron microscopy with *Xcc *surface-specific polyclonal antibodies indicates that the OMVs are released from the surface of *Xanthomonas *cells and therefore originate from the outer membrane. The LPS-specific SDS-PAGE confirms this finding, indicating that the OMVs contain LPS. Interestingly, however, they do not contain any or comparable amounts of LPS synthesis intermediates as could be displayed with whole cell LPS preparations. Therefore, it can be proposed that the OMVs are released from sites of the outer membrane that mainly consist of mature LPS.

### The OMV proteome does not contain all high abundant outer membrane proteins

The results from the proteome experiments also clearly indicate that the OMVs originate from the outer membrane. The comparison between the OMV and the most abundant OM proteins only permits a closer look at the outer membrane protein composition, since the OM fraction is contaminated with inner membrane fragments. Among the most abundant proteins in the OM fraction are 15 outer membrane proteins of which only eight are also present in the OMV proteome. The 11 non-outer membrane proteins are probably loosely associated with either the inner or outer membrane. Interestingly, three of these proteins could also be identified in OMVs. How proteins, that associate with the inner membrane like the signal recognition particle Ffh and the type-II secretion ATPase end up in the OMV fraction is unclear. Among these non-outer membrane proteins is also the elongation factor TufA. This protein is known to be released or leaked from cultures of numerous bacteria and can therefore be identified in many protein fractions [44]. However, TufA cannot be identified in the OMV protein fraction, indicating that the OMV release from the outer membrane is not an effect of damaged cells. Furthermore, no cytosolic proteins could be identified in the OMV fraction and only one inner membrane protein was present in the OMV sample. This is also an indication that these structures are not products of cell degradation, since we would expect a higher amount of proteins from the inner membrane and of cytosolic origin.

### OMVs isolated from M9 and XVM2 medium contain known virulence-associated proteins

A comparison of the OMV proteomes obtained from different growth conditions revealed a clear influence on the protein composition. Interestingly, the XVM2 medium did not elevate the amount of type-III secretion associated proteins. The only Hrp protein, which was not identified in the M9 sample was HrpXv, which is known to be the regulator of the *hrp-*operon and induced by XVM2 [27]. The adhesin XadA1 was also among the XVM2 induced proteins. It could play a role in the attachment of OMVs to the host surface as it has been described for lipopolysaccharides mimicking the lewis blood antigens present on the surface of OMVs from *Helicobacter pylori *[28]. The OMVs were also found to contain a vesicle specific core proteome of 7 proteins, which are not among the most abundant OM proteins. This group of proteins mainly consists of virulence associated proteins, which may indicate a putative role of these structures in the pathogenicity of *Xcc*. Virulence related proteins are also present in the group of proteins that differ between the cultivation conditions. Unexpectedly, we identified the so-called avirulence proteins AvrBs1 and AvrBs2, which are transported via the type-III secretion system [29,30]. Since they do not have trans-membrane segments [31] it can be assumes that they are located in the lumen of the OMVs. Another interesting OMV protein is HrpF, the putative translocon of the type-III secretion system. This protein is proposed to be inserted into the host membrane and serves as attachment site for the type-III conduit [32]. How this protein it transported into the membrane is not entirely clear. One model proposes, that HrpF is transported via the type-III system attached to the tip of the conduit and then pushed through the host membrane [11,12]. We offer a model in which HrpF is inserted into the bacterial outer membrane and then released into the medium packed in OMVs. These OMVs could then first attach to the host membrane and then fuse with it to insert HrpF. A similar mechanism has been described for the cytotoxic ClyA protein of enterotoxic *E. coli *strains. Subunits of this protein are inserted into the bacterial outer membrane and released into vesicles where they multimerise to give rise to the mature pore. Upon fusion of the OMV with the host membrane the cytotoxic pore is inserted and causes lysis of the host cell [16].

We could demonstrate that *Xcc *releases OMVs from its cell surface packed with virulence-associated proteins. Furthermore, a comparison with the most abundant proteins from the outer membrane revealed that some proteins are enriched in the OMV fraction. Further experiments will indicate if OMVs serve as vehicles for virulence factors like HrpF and avirulence proteins into the plant cell.

## Conclusion

The plant pathogenic bacterium *Xanthomonas campestris *pv. campestris liberates OMVs from its outer membrane during growth. We could demonstrate that these structures not only contain membrane-associated proteins, but also soluble periplasmic proteins, which are probably entrapped in the OMV lumen. A comparison with the most abundant proteins from the outer membrane fraction revealed, that not all high abundant proteins are released with OMVs, suggesting that some proteins are favourably released with these structures. This may be connected to the membrane composition of the OMVs, which differs from whole cell LPS preparations [33]. Nearly half of the proteins that associate with the OMV fraction are involved in virulence either being part of the TTSS, putative virulence factors or cellulytic enzymes. From the results presented in this study we conclude that plant pathogenic bacteria like *Xcc *can liberate membrane- and virulence-associated proteins attached to OMVs into the culture medium. Further experiments will have to prove if OMVs can attach and fuse with plant membranes. This would indicate whether OMVs from plant pathogenic bacteria can deliver virulence-factors as it has been demonstrated for mammalian pathogens.

## Methods

### Bacterial Strains and Growth Conditions

Bacterial cultures of *Xanthomonas campestris *pv. campestris strain B100 [34] were cultivated at 30°C. For inoculation of minimal media 10 mL TY cultures supplemented with 800 μg·mL^-1 ^streptomycin were inoculated with a single colony from fresh culture plates. The Minimal media (M9 or XVM2) [13,35,36] were always cultivated in 1 L Erlenmeyer flasks filled with 250 mL of medium and agitated at 150 rpm. The starting o.D. (580 nm) of the cultures in minimal media were adjusted to 0.01.

### Isolation of OMVs from culture supernatants

OMVs were isolated from culture supernatants using a method described by Wai *et al*. [37] with some modifications. The *Xcc *B100 cultures were harvested at o.D. (580 nm) 0.8–1.0. Cells were removed by centrifugation (5000·*g*, 30 min) and the supernatant was centrifuged at 10000·*g *for one hour and subsequently filtered through a 0.2 μm filter to remove residual cells. OMVs were recovered from the resulting supernatant by ultracentrifugation at 100000·*g *for 6 hours. The pellets were suspended in 100 μL rehydration buffer, 1% (v/v) lauroyl-sarcosine, 1% triton X-114 for subsequend SDS-PAGE. For electron microscopy, the vesicle pellet was resuspended in 50 mM HEPES buffer (pH-6.8).

### Hot-phenol extraction of LPS

The LPS was extracted as described earlier [38]. *Xcc *cells were grown for 3 days on TY plates and then washed from the surface with cold 0.9% (w/v) NaCl. The cells were recovered by centrifugation at 10000·*g *for 30 min at 4°C. The resulting pellet was washed twice with cold 0.9% (w/v) NaCl. The washed cells were resuspenddd in water, heated to 70°C and mixed with an equal volume of preheated water saturated phenol. After 45 min at 70°C the suspension was allowed to cool down on ice for 10 min prior to centrifugation at 10000·*g *for 60 min at 4°C. The water phase was dialysed (13000 MWCO, Roth, Karlsruhe Germany) against water untill all phenol was removed. The dialysed LPS was transferred to a 50 ml reaction tube and treated with 5 μl RNase (3 mg·ml^-1^) and 150 μl DNase (3 mg·ml^-1^) for 3 h at 37°C. Remaining proteins were removed with 150 μl Proteinase-K (2.5 mg·ml^-1^) and incubated over night at 37°C. The LPS extract was lyophilized prior to storage at -80°C.

### Crude LPS extraction from whole cells and OMVs

A few *Xcc *colonies (or OMV suspension) were transferred from a TY plate into 50 μl of lysis buffer (1 M Tris pH 6.8, 2% (w/v) SDS, 4% (v/v) β-mercaptoethanol, 10% (v/v) glycerol, 0.05% bromo-phenol-blue) and boiled for 10 min. Then 10 μl Proteinase-K was added and incubated at 37°C for 60 min. The remaining cells were removed by centrifugation at 13000 rpm in a biofuge and the supernatant was transferred into a fresh reaction tube. The crude LPS extract was mixed with 0.5 volumes of LPS-loading buffer (120 mM Tris pH 6.8, 3% (w/v) SDS, 9% (v/v) β-mercaptoethanol, 30% (v/v) glycerol, 0.003% (w/v) Bromo-Phenol-Blue) prior to gel electrophoresis.

### LPS-specific silver stain

The gels were fixed over night in fixing solution (40% (v/v) ethanol, 5 (v/v) acetic acid) replaced with oxidation solution (0.7% (w/v) periodic acid in fixing solution) and left to incubate for 5 min. The gels were then washed thrice with deionised water prior a 10 min incubation step in staining solution (140 ml deionised water, 2.8 ml 1 N NaOH, 5 ml 20% (v/v) AgNO_3_, 2 ml ammonia). After staining, the gels were washed thrice with deionised water and then placed into new containers with developer (50 μl citric acid (100 mg·ml^-1^), 50 μl formaldehyde in 100 ml water) an incubated untill bands became visible.

### Isolation of outer membrane proteins (OMPs)

*Xcc *cells were harvested from 250 mL of M9 culture by centrifugation at 5000·*g*, 4°C for 45 min. The pellet was washed twice with SM buffer (100 mM NaCl, 10 mM MgSO_4_, 20 mM Tris-HCl pH 7.5) and centrifuged as already described. After washing, the pellet was gently resuspended in 50 mM Tris-HCl pH 8.0, 1 mM EDTA and sonificated on ice 10 times for 6 seconds with 30 seconds resting time between each period in order to avoid overheating. Intact cells and debris were removed by centrifugation at 5000·*g *for 30 min at 4°C. The supernatant was then subjected to ultracentrifugation at 100000·*g *for 60 min at 4°C and the pellet containing proteins was washed in 10 mL Tris-HCl and 2% (v/v) lauroyl-sarcosine and incubated at 30°C for 30 min. The OMPs were collected by centrifugation at 10000·*g *for 90 min at 4°C. The resulting pellets were dissolved in 100 μL of rehydration buffer.

### SDS-PAGE Electrophoresis

The standard SDS-PAGE procedure was used [39]. 50 μg of protein sample was loaded onto a 12% (w/v) SDS-PAGE. The proteins separated by SDS-PAGE were stained with Coomassie Blue. Protein molecular weight standards were obtained from Bio-Rad (Munich, Germany).

### In-gel tryptic digest of proteins

The tryptic digest was performed as described on the Keck home page [45]. Protein sections were excised from SDS-PAGE gels and placed into a microtiter plate. 200 μL of 50% (v/v) acetonitrile (ACN) was added to each sample and incubated while shaking at room temperature (RT) for 5 min. Then this solution was completely removed and 200 μL 50 mM NH_4_HCO_3_, 50% (v/v) ACN was added to the samples and allowed to incubate for 30 min whilst shaking at RT. After complete removal of this solution the incubation step was repeated with 10 mM NH_4_HCO_3_, 50% (v/v) ACN. Then the samples were allowed to dry completely. Each sample was treated with 80 μL of previously activated solution of trypsin and incubated for 5–10 min. 100 μL of 10 mM NH_4_HCO_3 _was added to the samples and the samples were incubated at 37°C for 20–24 hours. The solution was removed next day and transferred to 1.5 mL eppendorf tubes pre-washed with 0.1% TFA. The peptides were extracted by adding 200 μL 0.1% TFA and 60% ACN with shaking at RT for 60 min. This step was repeated twice. The samples were then frozen at -80°C and lyophilized.

### Nano-LC-ESI-MS/MS measurement trypsin digested gel sections

Electrospray-ionisation-MS was performed on an LCQ_DECA_™ mass spectrometer (Thermo Fisher Scientific, Waltham, USA) with previously desalting and separation of the tryptic peptides by a NanoLC (Eksigent Technologies, Livermore, USA) run. Dry digests were reconstituted in 10 μl of 5% ACN in 0.1% TFA. The LC-system was run with solvents A = 5% and B = 80% ACN, respectively, in 0.1% formic acid. The tryptic peptides were subjected to the nano-column (15 cm × 75 μm i.d., PepMap™ C18, 100Å; Dionex, Idstein, Germany) by an autosampler device. First, for binding and desalting of the peptides a 5-min step of 98% solvent A/2% solvent B was run using a nano-flow of 200 nl·min^-1^. Subsequently, the peptides were eluted from the column applying a gradient with increasing acetonitrile concentration (2% – 50% 'B' in 40 min) with the same flow rate (200 nl·min^-1^). The separated peptides were transferred via a fused silica capillary (50 μm i.d.) to the nanospray needle (15 μm tip, New Objective, MA, USA) of the LCQ Deca ion source. Ionization and sample uptake by the mass spectrometer occurred with a spray/needle voltage of 1.3 kV and a capillary voltage of 30 V at 165°C. Via a MS to MS/MS switch mode (zoom scan + dependent scan) relevant precursor ions were detected automatically for MS/MS-acquisition using the X-calibur software™, (Thermo Fisher Scientific). Peptide fragmentation was initiated by collision energy in the ion-trap MS. For subsequent database search the entire MS/MS-spectra per run were converted into dta-files using the Bioworks 3.0 software (Thermo Fisher Scientific). The identification of the proteins was carried out using the Mascot software (Matrix Science, London, UK), which is implemented on our local server. The *Xcc *database generated from the *Xanthomonas campestris *pv. campestris B100 sequencing project [6] was used to perform the Mascot-aided search. The following parameters were used for the MS/MS ion search: enzyme: trypsin; missed cleavage: 2; peptide tolerance 150 ppm; M/MS tolerance: 100 ppm; peptide charge +1, +2 and +3; ion type: monoisotopic. For comparability, the accession numbers were taken from the *Xcc *strain ATCC 33913 database [3] as they are used by the expasy [46] internet resource.

### MALDI-TOF-MS measurements

Protein bands were excised from Coomassie stained acrylamide gels and digested with trypsin as already described. The supernatants of the in-gel digests were mixed with the same volume of a solution containing water, acetonitrile and TFA (67:33:0.1), which was saturated with α-cyano-4-hydroxy cinnamic acid. These solutions were spotted on to an Anchor-Chip™ (Bruker, Bremen) MALDI target and left to dry at room temperature. This spotting procedure was repeated thrice each time applying 1 μl of sample. The corresponding peptide mass fingerprints (PMF) were measured with an Ultraflex™II (Bruker, Bremen) MALDI-TOF-MS using the manufacturers settings for peptides measurements. The Mascot software was used for queries in our *Xcc *database (described above). The following parameters were chosen for the query: enzyme: trypsin; missed cleavages: 1; peptide tolerance: 150 ppm; mass values: MH^+ ^mono isotopic.

### N-terminal secretion signal-peptide prediction using the Signal P software

The Signal P software [40] was used to search the genome sequence of *Xcc *ATCC 33913 for proteins with putative N-terminal secretion signals. The following parameters were set to produce most reliable results: (a) organism type: Gram-negative, (b) only the first 60 N-terminal amino acids were submitted, (c) both, Neural Network and Hidden Markov Model algorithms must predict the same cleavage site, (d) the prediction score had to be higher than 0.98.

#### Electron Microscopy

Vesicles obtained by ultracentrifugation (100,000 × g, 6 hours) were resuspended in 50 μL of 50 mM HEPES buffer (pH 6.8). Nickel grids supported with formvar coating were used for electron microscopy. For this purpose, glass slides were cleaned with ethanol and then dipped in a formvar solution containing 0.6% formvar in chloroform. They were allowed to dry and then scratched carefully from the sides to loosen the film. The slide was lowered into the water at a 30 degree angle such that the formvar coating floats off onto the surface of the water. Using the forceps, the grids were placed in rows on the surface of the formvar with the shiny side facing up until the entire formvar sheet was covered with grids. The grids with formvar film adhering to them were picked using paraffin wax from the water surface and subsequently coated with carbon after placing them on a Whatman filter paper to dry. These carbon coated formvar grids were then used for observing outer membrane vesicles. 20 μL of purified OMVs were placed on glow-discharged carbon-coated formvar grids, which were subsequently fixed with 1% (v/v) glutaraldehyde, rinsed three times with water and visualized by staining with 1% (w/v) uranyl acetate. The size of the OMVs was determined by comparing with the width of the tobacco mosaic virus.

### Electron Microscopy of OMVs with gold-labelled anti-Xcc antibodies

Immunogold Electron Microscopy (IEM) was performed using fresh cultures of *Xcc *and OMVs prepared as described above. For IEM, carbon-coated formvar grids were used and the surface of the grid was prepared by covering it briefly with a 0.1% (w/v) solution of poly-L-lysine in water [41]. The surface of the grids was washed thoroughly with water and subsequently placed on a drop of bacterial culture or OMV suspension. The cells or OMVs were allowed to settle in order to adhere to the grids. The adhered bacteria or vesicles were preincubated for 30 min at RT with blocking buffer (0.1% normal goat serum) followed by three washing steps with phosphat saline buffer containing 0.1% (w/v) bovine serum albumin (BSA) (Sigma-Aldrich, Steinheim, Germany). The samples were subsequently incubated for 1 hour at RT on droplets containing an anti-*Xcc *B100 antibody (Eurogentec, Seraing, Belgium) [22] in a 1:50 dilution that is able to specifically recognise *Xanthomonas *LPS in the OMVs. Non-bound antibody was removed by washing the grids twice for 10 min on PBS containing 0.1% (w/v) BSA. The primary antibody was detected using goat-anti-rabbit antibody (Aurion, Wageningen, NL) coupled to 10 nm gold particles. The conjugates were diluted 1:20 in PBS containing 0.1% (w/v) BSA and incubated for 30 min at RT. Unbound conjugate was removed by a sequence of washing steps (twice with PBS containing 0.1% (w/v) BSA for 5 min; once with PBS for 3 min and a final wash with water for 3 min) at RT. Before negative staining with 1% (w/v) uranyl acetate, the grids were washed rapidly on four droplets of water. The preparations were analysed with Philips CM 100 (Philips Electronics, Eindhoven, NL) and Zeiss EM109 (Carl Zeiss Inc. Oberkochen, Germany).

## Authors' contributions

VKS carried out the electron microscopy, the proteome study and was involved in drafting of the manuscript. FJV was responsible for the annotation of the *Xcc *strain B100 genome, which was used in the proteome study. KN was involved in the design of the study and helped to draft the manuscript. SAW designed the study and was involved in drafting the manuscript. All authors have read the final version of the manuscript.

## Supplementary Material

Additional file 1 Tables 4-6. Click here for file
