# Supplementary material for: Analysis of outer membrane vesicle associated proteins isolated from the plant pathogenic bacterium Xanthomonas campestris pv. campestris
Source: BMC Microbiol. 2008 Jun 2;8:87. doi: 10.1186/1471-2180-8-87 (PMC2438364; doi:10.1186/1471-2180-8-87)
Supplement: Additional file 1 — Tables 4-6. [file 1471-2180-8-87-S1.doc]

Table 4. Additional MS/MS data belonging to the ESI-MS measurements listed in table 1

| **Section** | **Protein** | **Acc.No.** | **Score** | **No. of peptides matched** | **Matched Peptide sequences** | **Individual**  **Ion Score** |
| --- | --- | --- | --- | --- | --- | --- |
|  |  |  |  |  |  |  |
| 1 | TonB-dependent receptor with signalP | AAM 42139 | 162 | 11 | IDILK  FIETGGR  SFNQNVK  FDPQYDVPGR  GPPVAYSTSANR  QGVTSVADVLQR  ATADSELEQFQGR  NHIGATTYHDIQVR  TFNNGGDGSSGIALR  SADIETSQPVLTLTR  CYGGSAGEQAEYCALVTR | 19  13  55  61  46  33  28  74  100  35  53 |
|  | Outer membrane protein  With signalP | AAM40663 | 113 | 5 | GQPLGGSLK  FELGYGDSYGK  APVGPISISYAFPLK  APVGPISISYAFPLK  VGLETTLPGSTVEYYK | 19  15  22  51  60 |
|  | **Oar** putative outer  membrane receptor protein, tonB-dependent with  signalP | AAM41771 | 91 | 5 | FNLIR  IIDISR  LNDLLYIPAGR  EQIESLPSINR  SGTNEFHGSVYGTYR | 26  16  19  39  12 |
|  | TonB-dependent outer membrane receptor | AAM41663 | 49 | 4 | RKYFIER  LSAATVDYKVGYTK  YIGSTGLVSLGVFNK  RASDAIQDAVASDAMGR | 14  28  14  7 |
|  | **FpvA** TonB-dependent  outer membrane  ferripyoverdine receptor with signalP | AAM42628 | 59 | 5 | DQITPENTGNVPRR  SSVSVSTVGAEAIEQSAPR  SRVVAAWQDR  NWDGNASEVTYAR  LADPLSLIAGAR | 13  58  17  51  25 |
|  | **HrcV** (HrcV) | AAM40527 | 32 | 5 | GGNLTADEAR  ISSLGLDIGR  SILESLVVWGPK  DTSGSDQPALPSTSRK  LDPALLDQAIESERR | 16  18  23  26  32 |
|  | **HrpF** (HrpF protein) | AAM40515 | 31 | 9 | NLEAAGGVPLGK  NATYNPEKMK  SALQGLQKDPR  DDKADPAVRDAAK  TPVLAGLHYEEVR  TLDTPPTHAATSPEQK  TLDTPPTHAATSPEQK  GAAKGVAEGAAKGATQGTTK | 18  22  26  43  12  28  32  26 |
|  | **AcrD** Outer membrane efflux protein | AAM41427 | 27 | 7 | LEPVAGVAAVK  LDANPTESGENIGK  LAQLSLPIDNVITR  ERGLSVLDAAMVGTR  VWRGATRVVAPVMR  VGGGLEDEIQVDIDQQK  LTVVMAGGGSPAVEAAATER | 13  17  19  28  39  27  53 |
| 2 | **FadL** outer membrane  fatty acid porin with signalP | AAM39336 | 235 | 7 | MSETLTLR  MSETLTLR  MSETLTLR  LFQADVSAISFSAK  LFQADVSAISFSAK  VHGDVLAASINYKF  GGVAYDQTPTTAEHR | 28  56  20  9  80  28  72 |
|  | **Egl** exported cellulase with  signalP | AAM42791 | 140 | 6 | VVQLK  VIAEFNAR  FVANR  GSAAVLAVAPK  NVPYVLGLDLK  NADLQGLTSLQILDK | 29  16  14  24  12  67 |
|  | Avirulence protein with signalP | AAM43445 | 27 | 2 | NPEFGADLR  QAVPEITARPAGW | 28  17 |
|  | **HrcN** HrcN protein | AAM40534 | 30 | 2 | SVVVCATSDR  YGKVVEVIGTMLK | 19  26 |
|  | **Ffh** Signal recognition particle protein | AAM40492 | 27 | 4 | RAR  IKVR  FESLTQR  EVPRMIAIINSMTK | 13  26  17  14 |
|  | **AvrBs2** (avirulence protein) | AAM39371 | 27 | 2 | ARMR  TTAGAFR | 26  15 |
| 3 | **OmpA** family outer membrane protein with signalP | AAM40245 | 405 | 17 | VAVR  LSER  YPDLK  NGVDASR  AELAYR  TELNVQN  HFIAEGR  HFIAEGR  GTDAYNQK  RTELNVQN  RTELNVQN  VEVAGHTDSK  ATTVYDYLTK  AGVGLQTTFDKR  RATTVYDYLTK  LTNDAPFVTLGLGK  SEEEFDATPNPVSPGQQK | 17  25  38  24  25  27  38  40  28  39  43  53  23  32  20  61  32 |
|  | **AvrBs1** avirulence protein AvrBs1 | AAM41388 | 32 | 4 | DEAGSHIR  LLHVTNAGEA  SKMVADFIER  VLVQNLAQLQR | 12  18  26  28 |
|  | **XynB** exported xylan 1,4-beta-xylosidase with signalP | AAM43196 | 32 | 4 | LQVR  LQVRKTGYR  FYALSVGSDFPGTLIR  QDTKLSASPEAIVGDVR | 17  26  18  15 |
|  | **HrcU** HrcU protein | AAM40528 | 27 | 2 | KLFSLR  GVDEGALELR | 17  26 |
| 4 | **OmpA** family outer membrane protein with signalP | AAM40245 | 402 | 20 | LSER  YPDLK  NGVDASR  DGNFAAK  AELAYR  RYPDLK  RYPDLK  TELNVQN  HFIAEGR  GVNFDFNK  GVNFDFNK  RTELNVQN  VEVAGHTDSK  ATTVYDYLTK  ATTVYDYLTK  RATTVYDYLTK  LTNDAPFVTLGLGK  LTNDAPFVTLGLGK  ATTVYDYLTKNGVDASR  SEEEFDATPNPVSPGQQK | 20  23  26  17  42  61  32  43  27  52  23  12  19  62  53  22  42  48  21  15 |
|  | **VirB6** protein | AAM42571 | 32 | 3 | VIGAVAVAFLVK  DDEKTRAR  AALIIGLATSMAEGSSK | 28  17  41 |
|  | **Hrc U** HrcU protein | AAM40528 | 30 | 3 | GVDEGALELR  DNDEGSGPLPC  ALMSIGFDFATNTR | 26  29  9 |
|  | **RlpA** Exported rare lipoprotein A  With signalP | AAM42727 | 35 | 4 | EDPSTR  NSITGPK  YRVADSR  GLTEADNANLLAKRR | 5  12  14  17 |
|  | **HrpW** (HrpW protein) | AAM40517 | 28 | 1 | riagcrpev sgrpk | 32 |
| 5 | Putative exported protein | AAM40085 | 53 | 3 | FLDTPPGLHLK  VVLQHLLVDEK  SGANEVVIVVEDSPR | 60  66  94 |
|  | **OmpW3** OmpW family outer membrane protein | AAM39855 | 41 | 2 | IDAASTTGQHVGVDTAK  IDAASTTGQHVGVDTAK | 41  19 |
|  | **RlpB** Rare lipoprotein B with signalP | AAM41890 | 31 | 3 | RIDSVVR  NSLALPPDTPAVK RGLKAAGATLADEDAK | 12  28  42 |
|  | Lipoprotein precursor with signalP | AAM41856 | 26 | 2 | KVQVILQ RAMALLGTPY  KTALGIELPR VSRDIAR | 42  12 |
| 6 | **OmpW3** OmpW family  outer membrane protein | AAM39855 | 58 | 2 | IDAASTTGQHVGVDTAK  IDAASTTGQHVGVDTAK | 55  33 |
|  | **HrpB4** HrpB4 protein | AAM40532 | 29 | 3 | IDNTAR  IDNTARWLQR  IDNTARWLQR | 14  26  16 |
|  | **HpaH** Putative transglycosylase HpaH | AAM40539 | 29 | 2 | EMINK  EMINK | 26  15 |
| 7 | Putative exported protein with signalP | AAM40594 | 238 | 5 | DTAEEAKK  TAEDTAEAAK  TAEDTAEAAK  TAEDTAEAAKDTAEEAK  TAEDTAEAAKDTAEEAKK | 23  26  74  71  41 |
|  | **XpsH** General secretion pathway protein | AAM39979 | 47 | 3 | LRSAGKAIAAQLR  VDVGWITGEVRSGPLR  DARWRVDVGWITGEVR | 38  28  8 |
|  | **UptD** outer membrane protein with signalP | AAM39910 | 39 | 2 | AAADADLAKAR  QVPQLALRAAADADLAK | 29  18 |
|  | **HrpE** HrpE protein | AAM40519 | 30 | 2 | KNELDFNVALNK  LGQARDLLGSDLSSR | 32  15 |

Table 5. Additional MS/MS data belonging to the ESI-MS measurements listed in table 2

| **Section** | **Protein** | **Acc.No.** | **Score** | **No. of peptides matched** | **Matched peptide sequence** | **Individual Ion scores** |
| --- | --- | --- | --- | --- | --- | --- |
| 1 | TonB-dependent outer membrane receptor with signalP | AAM 42139 | 664 | 23 | FGFR  IDILK  NLGSAR  NLGSAR  FIETGGR  SFNQNVK  SFNQNVK  TLVLVNGR  VTADGAEPR  GNYSEGFR  FDPQYDVPGR  FDPQYDVPGR  FDPQYDVPGR  GPPVAYSTSANR  QGVTSVADVLQR  QGVTSVADVLQR  ATADSELEQFQGR  NHIGATTYHDIQVR  TFNNGGDGSSGIALR  QAYDFTLGTTTDR  SADIETSQPVLTLTR  SADIETSQPVLTLTR  CYGGSAGEQAEYCALVTR | 23  32  36  12  16  15  45  22  24  33  8  27  34  26  21  61  33  11  29  44  75  23  55 |
|  | **Oar** putative outer membrane receptor protein, tonB-dependent with signalP | AAM41771 | 295 | 13 | DFSLK  FNLIR  DYSAVR  IIDISR  ATTDASGR  GTGSLVDAR  YDEPMVK  NLQDYVR  LGLIYQGR  MGTGTNVTR  LNDLLYIPAGR  EQIESLPSINR  SGTNEFHGSVYGTYR | 22  13  19  28  33  43  21  12  41  9  12  52  27 |
|  | TonB-dependent outer membrane receptor (C-terminal fragment) with signalP | AAM43353 | 271 | 10 | FLLGGK  DYPFVK  VITSNYK  FTNQDLR  NLFEQLALESVK  NLFEQLALESVK  VLAYDGLVMSLVNR  VLAYDGLVMSLVNR  AFDNPGQVPVVESPSK  AFDNPGQVPVVESPSK | 11  18  36  37  21  19  16  41  21  46 |
|  | TonB-dependent outer membrane receptor (C-terminal fragment) with signalP | AAM42431 | 141 | 9 | ADAVVTR  YYAGLR  YYAGLR  VPGVQIR  SLYYFK  SLYYFK  GFQFGIR  NPALPPDAVLFLNGR  VTGLGSQGLPAINPQVMK | 22  46  15  16  17  42  61  72  36 |
|  | TonB-dependent outer membrane receptor with signalP | AAM42675 | 53 | 4 | LTNLGSIK  TSYAEGFR  TSYAEGFR  TAELQGQVPIQTLNR | 25  16  46  24 |
| 2 | TonB-dependent outer membrane receptor with signalP | AAM42139 | 630 | 15 | FIETGGR  SFNQNVK  FDPQYDVPGR  GPPVAYSTSANR  QGVTSVADVLQR  ATADSELEQFQGR  NHIGATTYHDIQVR  TFNNGGDGSSGIALR  SADIETSQPVLTLTR  CYGGSAGEQAEYCALVTR | 18  28  71  34  11  9  21  28  17  61 |
|  | TonB-dependent outer membrane receptor with signalP | AAM42044 | 628 | 14 | VMVGLR  YTEALR  YTEALR  ELVQTPR  VVAGVAFSK  EYYTSIR  WDVLNER  ETYYGYR  TASLTGDPIAGR  DTPQTITVVTK  GFTASSDITTDGVR  QTMDQQNLLSLR  VDGIALGAAGYLTER  ACVGQPPSTWNPNSGPR | 33  21  15  11  67  72  12  11  34  26  33  22  24  52 |
|  | **FpvA** TonB-dependent ferripyoverdine receptor precursor with signalP | AAM42628 | 506 | 17 | IEVIR  YHDTK  VNAAHTK  GVYASTR  GNVQSLR  FVSVSLR  LPGAWER  LSSWETR  DYYYDR  VVAAWQDR  IGEDWLLR  TQAFDASGAYTGTSGR  DQITPENTGNVPRR  SSVSVSTVGAEAIEQSAPR  SRVVAAWQDR  NWDGNASEVTYAR  LADPLSLIAGAR | 21  14  42  36  28  16  27  33  12  24  51  62  27  18  10  28  17 |
|  | **PhuR** outer membrane hemin receptor, tonB-dependent with signalP | AAM41930 | 317 | 16 | LPMIR  VDYFR  GGELGLR  FGAVWR  VDDIFR  LQVTATR  FTGASGVR  HSYVGLK  AIDTGTLR  NVDDVVIK  NFVDLETLK  EQLDNQLVR  LTVEGNEDDGR  NFVDLETLKR  DGYSLNLANGAVSK  GPASSLYGSDALGGVVAFVTK | 11  75  25  32  21  11  63  14  44  37  29  8  31  25  23  69 |
|  | TonB-dependent outer membrane receptor with signalP | AAM42316 | 218 | 7 | YAFTDK  FSAGLAGR  IDFHTR  VALRGTVSSGFR  IVLSSNLTGTGVR  GLSPDQVLVLVNGK  SVAANPAALSANGLSLER | 32  25  18  12  54  61  27 |
|  | Outer membrane protein with signalP | AAM40663 | 113 | 5 | GQPLGGSLK  GDTVDDGKVADSIR  APVGPISISYAFPLK  APVGPISISYAFPLK  VGLETTLPGSTVEYYK | 54  36  23  19  14 |
|  | **Bgl** exported beta-glucosidase with signalP | AAM41065 | 34 | 3 | DQGDTK  LGLFEAGKPSKRPLGGK  RDSAVTAQVWLGVGCGEK | 34  26  18 |
| 3 | **Egl** exported cellulose with signalP | AAM42791 | 142 | 6 | VVQLK  VIAEFNAR  FVANR  GSAAVLAVAPK  NVPYVLGLDLK  DKTWQDALVK | 24  36  17  11  28  38 |
|  | OmpA family outer membrane protein with signalP | AAM40245 | 98 | 4 | TELNVQN  DGNFAAK  AELAYR  RYPDLK | 46  39  24  22 |
|  | **Ffh** Signal recognition particle protein | AAM40492 | 31 | 6 | IKVR  IKVR  NPALLNGSR  ERRNPALLNGSR  SLTPGQALIKIVR  GGAALSVRYITGKPIK | 35  18  22  27  16  9 |
| 4 | **OmpA** family outer membrane protein with signalP | AAM40245 | 581 | 23 | LSER  LSER  YPDLK  AELAYR  TELNVQN  DGNFAAK  AELAYR  RYPDLK  HFIAEGR  HFIAEGR  GVNFDFNK  GVNFDFNK  RTELNVQN  RTELNVQN  VEVAGHTDSK  VEVAGHTDSK  ATTVYDYLTK  ATTVYDYLTK  ATTVYDYLTK  RATTVYDYLTK  LTNDAPFVTLGLGK  LTNDAPFVTLGLGK  SEEEFDATPNPVSPGQQK | 52  13  26  32  19  17  24  23  12  10  32  16  49  10  38  32  16  26  13  22  24  62  17 |
|  | **FadL** outer membrane fatty acid porin with signalP | AAM39336 | 229 | 7 | MSETLTLR  MSETLTLR  DVRVPDASRK  LFQADVSAISFSAK  LFQADVSAISFSAK  VHGDVLAASINYKF  GGVAYDQTPTTAEHR | 27  35  17  11  28  33  14 |
|  | **TufA** (elongation factor Tu) | AAM40191 | 70 | 4 | LVEALDTFIPDPTR  LVEALDTFIPDPTR  LLDQGQAGDNAGLLLR  LALEGDQSDIGVPAILK | 26  66  17  14 |
| 5 | **OmpA** family outer membrane protein with signalP | AAM40245 | 402 | 20 | NGVDASR  AELAYR  TELNVQN  DGNFAAK  AELAYR  RYPDLK  HFIAEGR  HFIAEGR  GVNFDFNK  GVNFDFNK  RTELNVQN  RTELNVQN  VEVAGHTDSK  VEVAGHTDSK  ATTVYDYLTK  ATTVYDYLTK  RATTVYDYLTK  LTNDAPFVTLGLGK  LTNDAPFVTLGLGK  SEEEFDATPNPVSPGQQK | 21  32  23  32  19  15  54  23  62  17  32  16  29  17  38  32  19  26  33  14 |
|  | Putative secreted protein | AAM39862 | 41 | 4 | GSDGLIGLK  GSDGLIGLK  LIFDYFK  LIFDYFK | 44  36  15  10 |
|  | **Egl** exported cellulose | AAM42791 | 39 | 4 | VVQLK  YGEGDAR  NADLQGLTSLQILDK  DMIVQMQGLGFNAVR | 67  14  33  32 |
|  | **XpsE** Type II secretory pathway ATPase | AAM39976 | 37 | 4 | LKVR  LKVR  ATAAAPTGYLGR  VDGVLVEGESPPAK | 27  11  28  31 |
|  | **FliC** Flagellin A (Flagellin core protein) | AAM41230 | 31 | 4 | VASAINDK  VASAINDK  SRIADTDYAK  IRELSVQSANATNSATDR | 54  22  17  19 |
| 6 | **OmpA** family outer membrane protein with signalP | AAM40245 | 515 | 24 | LSER  YPDLK  NGVDASR  NGVDASR  AELAYR  TELNVQN  DGNFAAK  AELAYR  RYPDLK  HFIAEGR  HFIAEGR  GVNFDFNK  GVNFDFNK  RTELNVQN  RTELNVQN  VEVAGHTDSK  VEVAGHTDSK  ATTVYDYLTK  ATTVYDYLTK  ATTVYDYLTK  RATTVYDYLTK  LTNDAPFVTLGLGK  LTNDAPFVTLGLGK  SEEEFDATPNPVSPGQQK | 36  18  46  22  19  16  24  23  12  12  32  16  29  21  48  32  16  26  13  21  24  52  17  35 |
|  | Putative secreted protein | AAM39862 | 40 | 1 | LIFDYFK | 32 |
|  | **FadL** (outer membrane fatty acid porin) | AAM39336 | 26 | 2 | MSETLTLR  LFQADVSAISFSAK | 18  8 |
| 7 | **OmpA** family outer membrane protein with signalP | AAM40245 | 88 | 4 | YPDLK  DGNFAAK  AELAYR  RYPDLK | 22  15  34  17 |
|  | Putative exported protein | AAM40085 | 60 | 3 | FLDTPPGLHLK  FLDTPPGLHLK  VVLQHLLVDEK | 42  16  36 |
|  | **UptE** Outer membrane protein with signalP | AAM39911 | 37 | 6 | DDPQADALTR  DDPQADALTR  DDPQADALTR  MRQAAEQETLAR  VTIVGYDSDAATAK  VTIVGYDSDAATAK | 34  17  21  24  51  19 |
| 8 | **OmpW3** ompW family outer membrane protein | AAM39855 | 55 | 2 | IDAASTTGQHVGVDTAK  IDAASTTGQHVGVDTAK | 42  13 |
|  | **XpsH** General secretion pathway protein | AAM39979 | 47 | 3 | LRSAGKAIAAQLR  DARWRVDVGWITGEVR  DARWRVDVGWITGEVR | 12  15  20 |
|  | Putative secreted protein | AAM39862 | 41 | 2 | GSDGLIGLK  LIFDYFK | 41  10 |
| 9 | **UptD** outer membrane protein with signalP | AAM39910 | 57 | 3 | AAADADLAKAR  AAADADLAKAR  QVPQLALRAAADADLAK | 22  17  42 |
|  | **WxcE** exported protein | AAM39921 | 26 | 3 | DFLR  APEAEQPVK  APEAEQPVK | 34  17  44 |
| 10 | Putative exported protein with signalP | AAM40594 | 117 | 5 | DTAEEAKK  DTAEEAKK  TAEDTAEAAK  TAEDTAEAAK  TAEDTAEAAKDTAEEAK | 31  18  26  51  21 |
|  | **UptD** outer membrane protein with signalP | AAM39910 | 33 | 2 | AAADADLAKAR  QVPQLALRAAADADLAK | 57  31 |

Table 6. Additional MS/MS data belonging to the ESI-MS measurements listed in table 3

| **Section** | **Protein** | **Acc.No.** | **Score** | **No. of peptides matched** | **Matched peptide sequence** | **IndividualIon scores** |
| --- | --- | --- | --- | --- | --- | --- |
| 1 | TonB-dependent outer membrane receptor with signalP  **XadA1** Xanthomonas  adhesin XadA  **HrpA** ATP-dependent  RNA helicase  **RhsD** (RhsD protein) with  signalP | AAM 42139  AAM39974  AAM42217  AAM39448 | 311  35  32  32 | 9  3  8  3 | FGFR  IDILK  NLGSAR  FIETGGR  SFNQNVK  VTADGAEPR  ATADSELEQFQGR  QAYDFTLGTTTDR  SADIETSQPVLTLTR  QVANVAAGTR  ATAAGAGATASGAR  ATAAGAGATASGAR  RASLSGVILR  GDVLMFLPGER  WQGKPGNPQLR  DLEGLRARFGER  IRYVVDPGYARVK  LHIEPISQASANQR  FGERAGQAFAARAGR  TVNDAIVAAIDEITR  Kiangwr  Sylyededfyr  ragaavmnyryngrgqqvr | 26  35  21  42  33  45  33  62  76  23  26  12  42  15  22  12  33  61  33  9  19  29  12 |
| 2 | **FpvA** TonB-dependent  outer membrane ferripyoverdine receptor with  signalP  TonB-dependent outer  membrane receptor  **HrpF** HrpF protein  **AvrBs2** (avirulence protein) with  signalP  **FasD** outer membrane  usher protein FasD | AAM42628  AAM41663  AAM40515  AAM39371  AAM40677 | 162  61  35  33  30 | 7  2  8  4  2 | YHDTK  VNAAHTK  LPGAWER  VVAAWQDR  SSVSVSTVGAEAIEQSAPR  SRVVAAWQDR  NWDGNASEVTYAR  LSAATVDYKVGYTK  YIGSTGLVSLGVFNK  NLEAAGGVPLGK  NATYNPEKMK  SALQGLQKDPR  DDKADPAVRDAAK  TPVLAGLHYEEVR  TLDTPPTHAATSPEQK  TLDTPPTHAATSPEQK  GAAKGVAEGAAKGATQGTTK  ARMR  ARMR  TTAGAFR  TTAGAFR  MVRLTNAPCW PR  KGMLLVTP LQSWQR | 32  22  64  71  12  33  16  42  28  32  13  16  22  45  42  11  8  21  33  42  12  26  21 |
| 3 | **OmpA** family outer membrane protein with signalP  **FadL** outer membrane  fatty acid porin with signalP  **XynB** exported xylan 1,4-beta-xylosidase with signalP  **UptE** Outer membrane protein with signalP  **AvrBs1** avirulence protein AvrBs1 | AAM40245  AAM39336  AAM43196  AAM39911  AAM41388 | 505 | 24  8  5  6  3 | LSER  YPDLK  YPDLK  NGVDASR  AELAYR  TELNVQN  DGNFAAK  AELAYR  RYPDLK  HFIAEGR  HFIAEGR  GVNFDFNK  GVNFDFNK  RTELNVQN  RTELNVQN  VEVAGHTDSK  VEVAGHTDSK  ATTVYDYLTK  ATTVYDYLTK  ATTVYDYLTK  RATTVYDYLTK  LTNDAPFVTLGLGK  LTNDAPFVTLGLGK  SEEEFDATPNPVSPGQQK  MSETLTLR  MSETLTLR  MSETLTLR  DVRVPDASRK  LFQADVSAISFSAK  LFQADVSAISFSAK  VHGDVLAASINYKF  GGVAYDQTPTTAEHR  LQVR  LQVRKTGYR  LQVRKTGYR  FYALSVGSDFPGTLIR  NDPLSLYIDMGMPK  LQVSGTK  DDPQADALTR  MRQAAEQETLAR  VTIVGYDSDAATAK  VQAQIQAEEAERMR  YERLQAQQTVAALAEAK  DEAGSHIR  SKMVADFIER  VLVQNLAQLQR | 32  12  26  32  19  16  24  23  12  10  32  16  29  8  38  32  16  26  13  11  24  42  17  65  66  12  32  42  37  12  41  19  55  42  13  58  66  44  16  12  75  25  15  33  28  16 |
| 4 | **OmpA** family outer membrane protein with signalP  **Egl** exported cellulose with  signalP  **Hrc U** HrcU protein  **HrpXv** AraC-type transcriptional regulator HrpX  **HrcN** HrcN protein | AAM40245  AAM42791  AAM40528  AAM40465  AAM40534 | 113  66  30  30  34 | 20  5  3  3  3 | LSER  YPDLK  DGNFAAK  DGNFAAK  AELAYR  RYPDLK  RYPDLK  TELNVQN  HFIAEGR  GVNFDFNK  GVNFDFNK  RTELNVQN  VEVAGHTDSK  ATTVYDYLTK  ATTVYDYLTK  RATTVYDYLTK  RATTVYDYLTK  LTNDAPFVTLGLGK  ATTVYDYLTKNGVDASR  SEEEFDATPNPVSPGQQK  QIVDDSGK  GSAAVLAVAPK  DKTWQDALVK  NVPYVLGLDLK  NADLQGLTSLQILDK  GVDEGALELR  GVDEGALELR  ALMSIGFDFATNTR  QEEAEDAYRQASK  SAALNCFAQLAMDR  KALTHFHLGQSTLALQTLER  SVVVCATSDR  LAEMPLLQTTLER  GTQCDVNVIVLIGER | 65  32  36  13  55  23  36  17  22  17  38  22  26  29  12  34  15  18  16  9  22  31  18  8  18  32  16  41  44  52  14  37  12  18 |
| 5 | **ompW3** OmpW family outer membrane protein  **HrpB4** HrpB4 protein | AAM39855  AAM40532 | 55  36 | 2  2 | RVNADGVGK  ISGQYHFGQA DNVFR  IDNTAR  IDNTARWLQR | 28  19  35  16 |
| 6 | **ompW3** OmpW family outer membrane protein  **XpsH** General secretion pathway protein | AAM39855  AAM39979 | 48  43 | 4  2 | RVNADGVGK  RVNADGVGK  ISGQYHFGQA DNVFR  IDAASTTGQHVGVDTAK  LRSAGKAIAAQLR  DARWRVDVGWITGEVR | 26  22  8  18  63  12 |
| 7 | Putative exported protein with signalP  **UptD** outer membrane protein  with signalP | AAM40594  AAM39910 | 245  27 | 8  2 | DTAEEAKK  TAEDTAEAAK  TAEDTAEAAK  TAEDTAEAAK  TAEDTAEAAKDTAEEAK  TAEDTAEAAKDTAEEAK  TAEDTAEAAKDTAEEAK  TAEDTAEAAKDTAEEAK  AAADADLAKAR  QVPQLALRAAADADLAK | 42  33  27  13  75  24  15  18  28  26 |
